# Age-related effects of high protein diet on rat liver and kidney

**DOI:** 10.1007/s11845-025-04265-z

**Published:** 2026-01-17

**Authors:** Fatma Helvacıoğlu, Eda Özturan Özer, Caner Çetinoğlu, Pınar Ülgen, Sera Kılıç, Deniz Can Başaran, Berk Yasin Ekenci, Fırat Yıldırım, Mustafa Agah Tekindal, Attila Dağdeviren

**Affiliations:** 1 Department of Histology and Embryology, Faculty of Medicine, Baskent University, Ankara, Turkey; 2 Department of Medical Biochemistry, Faculty of Medicine, Baskent University, Ankara, Turkey; 3 GFO Kliniken Troisdorf, Allgemein- und Viszeralchirurgie, Troisdorf, Nordrhein-Westfalen, Deutschland; 4 Gulhane Training and Research Hospital, Gynecology and Obstetrics Clinic, Ankara, Turkey; 5 Department of Dermatology, Ankara Bilkent City Hospital, Ankara, Turkey; 6 Department of Cardiovascular Surgery Department of Cardiovascular Surgery, Faculty of Medicine, Ege University, Izmir, Turkey; 7 Department of Urology, University of Health Sciences, Ankara Etlik City Hospital, Ankara, Turkey; 8 Department of Emergency Medicine, Burhaniye State Hospital, Balıkesir, Turkey; 9 Department of Biostatistics, Katip Celebi University School of Medicine, Izmir, Turkey; 10 Department of Histology and Embryology, Baskent University School of Medicine, Ankara, Turkey

**Keywords:** High protein diet, Liver, Kidney, Electron microscopy, MDA, GSH

## Abstract

**Backround:**

While low-carbohydrate/high-protein diets are common for rapid weight loss, their controversial side effects warrant investigation.

**Aims:**

This study aimed to examine age- and duration-dependent effects of high-protein diets on the liver and kidneys of adult (6 months) and elderly (18 months) rats.

**Methods:**

Thirty-two male Wistar albino rats were divided into four groups: Adult Standard, Adult High-Protein, Elderly Standard, and Elderly High-Protein. After one month, we collected kidney and liver samples for histological analysis and biochemical assessment of MDA and GSH levels.

**Results:**

High-protein diets severely affected the kidneys of both adult and elderly rats. The livers also exhibited moderate degenerative changes. We found a significant increase in MDA levels, indicating lipid peroxidation. Additionally, hepatic and renal GSH levels significantly increased in elderly rats on a high-protein diet, suggesting a metabolic response to oxidative stress.

**Conclusion:**

Our findings suggest that high-protein diets should be applied very cautiously, especially in elderly individuals and those with existing kidney disorders.

**Supplementary Information:**

The online version contains supplementary material available at 10.1007/s11845-025-04265-z.

## Introduction

Obesity and overweight are recognized by the World Health Organization as global epidemics that pose a major threat to public health and require urgent management [1]. The prevalence of obesity continues to rise, and modifying dietary habits, lifestyle behaviours, and health awareness remains a significant challenge. While governments and health authorities are increasingly alarmed, effective management also requires active involvement of healthcare professionals, including dietitians, nutritionists, and nutritional therapists [1, 2].

A variety of approaches are used to promote weight reduction. In recent years, low-calorie/high-protein diets have gained popularity among individuals seeking rapid and effective weight loss. Several studies have reported favorable outcomes, particularly in overweight and obese individuals, supporting the role of high-protein diets in weight control [3, 4]. However, concerns have also been raised regarding their long-term safety, with some evidence suggesting adverse effects on metabolic and organ health [5–7].

Given the ongoing debate and the conflicting evidence in the literature, further investigation is warranted. Although numerous experimental studies have investigated the effects of high-protein diets on hepatic and renal morphology as well as oxidative stress markers, few have explicitly compared how aging modulates these responses. Aging is associated with reduced antioxidant defenses, impaired mitochondrial function, and altered cellular stress regulation, which may increase susceptibility to diet-induced tissue injury. Therefore, the present study was designed to test the hypothesis that aging amplifies the structural and biochemical effects of a high-protein diet on liver and kidney tissues. By framing the adult versus elderly comparison as the central focus, this work aims to provide novel insights into the age-dependent mechanisms underlying organ responses to high-protein feeding.

## Materials and methods

Thirty-two male Wistar albino rats, comprising adult (6 months) and elderly (18 months) groups, were maintained under 12-hour light-dark cycles at 23 ± 2 °C with ad libitum access to water. Rats were randomly assigned to four groups (*n* = 8 per group): Adult Standard Diet (Group 1), Adult High-Protein Diet (Group 2), Elderly Standard Diet (Group 3), and Elderly High-Protein Diet (Group 4). Experimental groups received a high-protein diet (47.5% protein), while control groups received standard chow (20% protein) for one month. Body weights and food intake were recorded regularly. Diets were supplied by MBD Feed Trading Company (Gebze, Kocaeli).

An a priori power analysis using G*Power determined that a total of 32 rats (8 per group) provided 83.3% power for one-way ANOVA with a partial η² of 0.50 at a 5% significance level.

At the end of the experiment, rats were euthanized under general anesthesia (150 mg/kg ketamine), and liver and kidney tissues were dissected and weighed. Samples were fixed in 2% glutaraldehyde, processed for plastic embedding, and sectioned using an ultramicrotome. Semi-thin Sect. (500 nm) were stained with 1% toluidine blue and examined under a light microscope (Leica DM 3000, DFC500 camera). Ultrathin Sect. (70 nm) were contrasted with lead citrate/uranyl acetate and examined using a Carl Zeiss EVO LS 10 electron microscope.

istological assessment of structural alterations in the liver and kidney was performed using validated semi-quantitative scoring systems (0–4) in a blinded manner. Liver sections were evaluated for parenchymal injury using a 0–4 grading system based on the severity of hepatocytic vacuolization, sinusoidal dilatation/congestion, and cellular degenerative changes. For each sample, multiple fields from the hepatic lobules were examined at 400× magnification, and the final liver injury score was calculated as the average of all evaluated areas. In this system, a score of 0 indicates normal hepatic morphology; 1 + reflects minimal alterations such as mild vacuolization or slight sinusoidal widening; 2 + denotes moderate injury with more apparent vacuolization and sinusoidal dilatation; 3 + represents marked parenchymal damage characterized by widespread vacuolization and pronounced sinusoidal changes; and 4 + corresponds to severe injury with extensive cytoplasmic degeneration and diffuse structural disruption. The semi-quantitative liver injury scoring system was adapted from previously published histopathological evaluation approaches described by Menke et al. and Lo & Kim, with modifications based on the specific morphological features observed in our samples [8, 9].

Semi-thin sections were also evaluated for the severity of glomerular injury. The Glomerular Damage Index (GDI) was scored on a scale from 0 to 4 based on the extent of glomerulosclerosis, mesangiolysis, and mesangial expansion. For each section, 80–100 glomeruli from the renal cortex were examined, and the final GDI value was calculated as the mean score of all assessed glomeruli. Scoring was performed on toluidine blue–stained slides under 400× magnification. In this system, a score of 0 indicates the absence of lesions; 1 + corresponds to sclerosis affecting < 25% of the glomerulus; 2 + reflects 25–50% involvement; 3 + represents 50–75% sclerosis; and 4 + denotes sclerosis involving > 75% of the glomerular structure [10].

### Biochemical analysis

Liver and kidney tissue homogenates (10% w/v) were prepared in ice-cold 0.15 M KCl using an all-glass homogenizer to determine malondialdehyde (MDA) and glutathione (GSH) levels. MDA, a marker of lipid peroxidation and oxidative stress, was measured by adding two volumes of thiobarbituric acid reagent to one volume of homogenate, followed by incubation in a boiling water bath for 15 min, cooling, and centrifugation at 1000 × g for 10 min. Absorbance was measured at 535 nm using a spectrophotometer (Shimadzu UV-1601, Japan), and results were expressed as nmol MDA per gram of tissue.

GSH, an indicator of total antioxidant capacity, was measured after deproteinizing the samples. Ellman’s reagent was added, and absorbance was read immediately at 412 nm. Concentrations were calculated using a GSH standard curve and expressed as µmol GSH per gram of tissue.

### Statistical analysis

Data were analyzed using SPSS 25 (IBM Corp., 2017) and are presented as mean ± standard deviation or as frequencies. Normality and homogeneity of variances were assessed using the Shapiro–Wilk and Levene’s tests. For normally distributed data with homogeneous variances, paired t-tests were used for two-group comparisons, and one-way ANOVA was applied for comparisons involving three or more groups. Non-parametric alternatives included the Wilcoxon and Kruskal–Wallis tests for dependent and independent groups, respectively. Ordinal data, including the Glomerular Damage Index (GDI) and Liver Injury Scores, were analyzed using the Kruskal–Wallis test, followed by pairwise Mann–Whitney U tests with Bonferroni correction. These tests were selected because the scoring data are ordinal and may not meet the assumptions of normality. Repeated measures were analyzed using Repeated Measures ANOVA, applying Greenhouse–Geisser or Huynh–Feldt corrections as appropriate. Statistical significance was set at *p* < 0.05.

## Results

### Histological findings

**Fig. 1:**
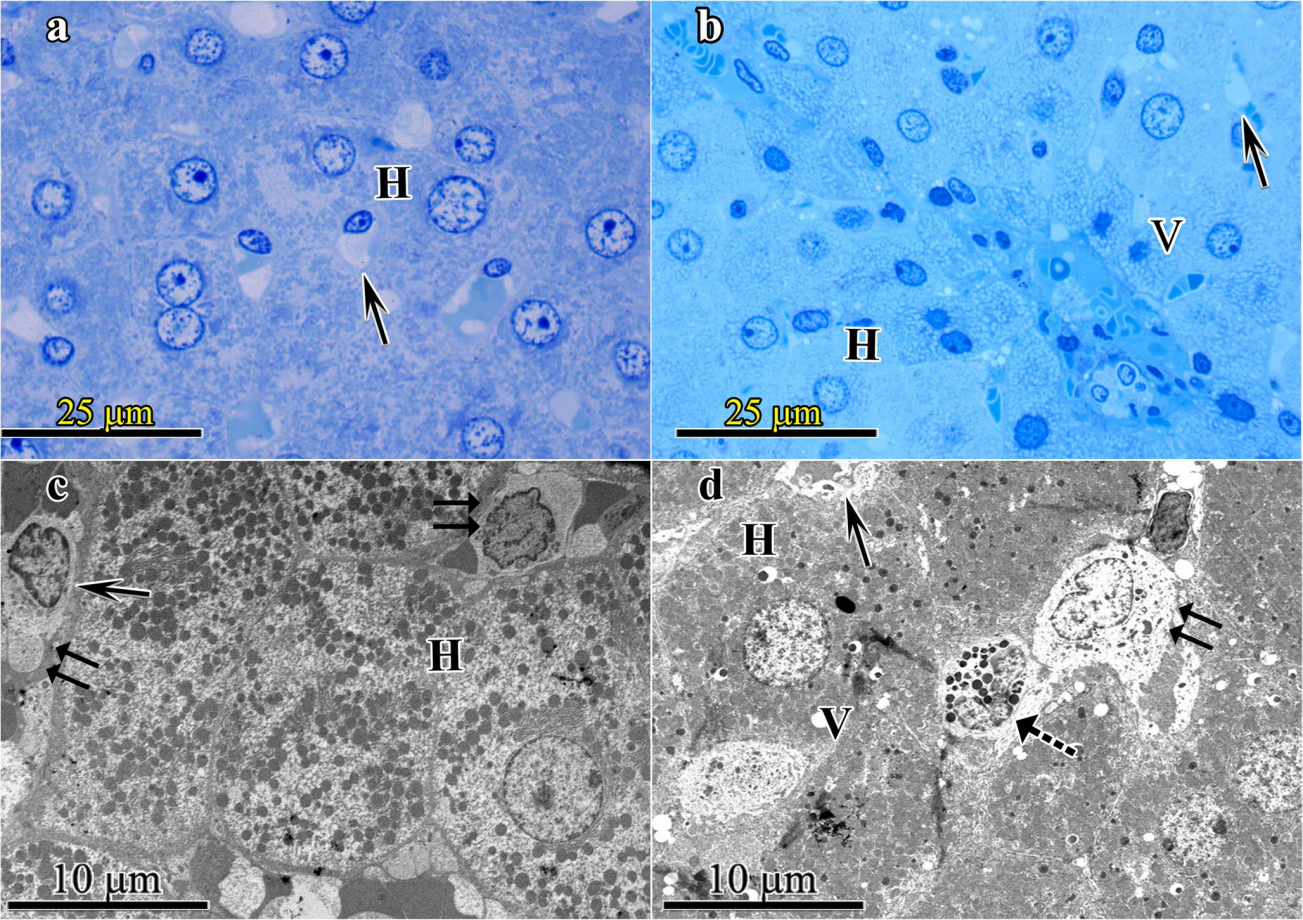
Liver- Adult groups fed with standard diet (Group-1) Fig. 1a and 1c; fed with high protein diets (Group-2) 1b and 1d; 1a; Group I - At light microscopic level the hepatocytes (H) and the sinusoids (→) between them are seen as normal. 1b; Group-2- tissue samples, at light microscopic level hepatocytes (H), vacuoles (V) and sinusoids (→) are observed. 1c; Group-I at electron microscope, hepatocytes (H), Kupffer cell (⇉), Sinusoids (→) are reflected normal structural features. 1d; Group-2 at ultrastructural level: Hepatocytes (H) have varying size and number vacuoles (V) are seen reflecting steatosis. In dilated liver sinusoids (→), Ito (⇢) and Kupffer cells (⇉) with a few heterophagosomes were exhibiting normal structural features. (1a and 1b: semi-thin section stained with toluidine blue; original magnification X100; 1c and 1d: thin sections stained with uranyl acetate-lead citrate; original magnification 1c: X5090; 1d: X3760)

Liver- Adult groups fed with standard (Adult SD (Group-1)) and high protein (Adult HPD (Group-2)) diets: In the examination of the livers belonging to Group-1 normal histological features of liver at both light and electron microscopic levels were seen. No structural degenerative change was observed in hepatocytes and liver sinusoids. Under electron microscope all organelles and nuclei of liver cells reflected normal structural features (Fig. 1a and c). In Group-2 tissue samples, mild swelling in hepatocytes with vacuoles of varying sizes and numbers in the cytoplasm were detected at light microscopic level. Dilated liver sinusoids along with associated cells were also observed. Under electron microscope distinct degenerative changes in hepatocytes and endothelial cells were detected. Ito and Kupffer cells were exhibiting normal structural features (Fig. 1b and d).

**Fig. 2:**
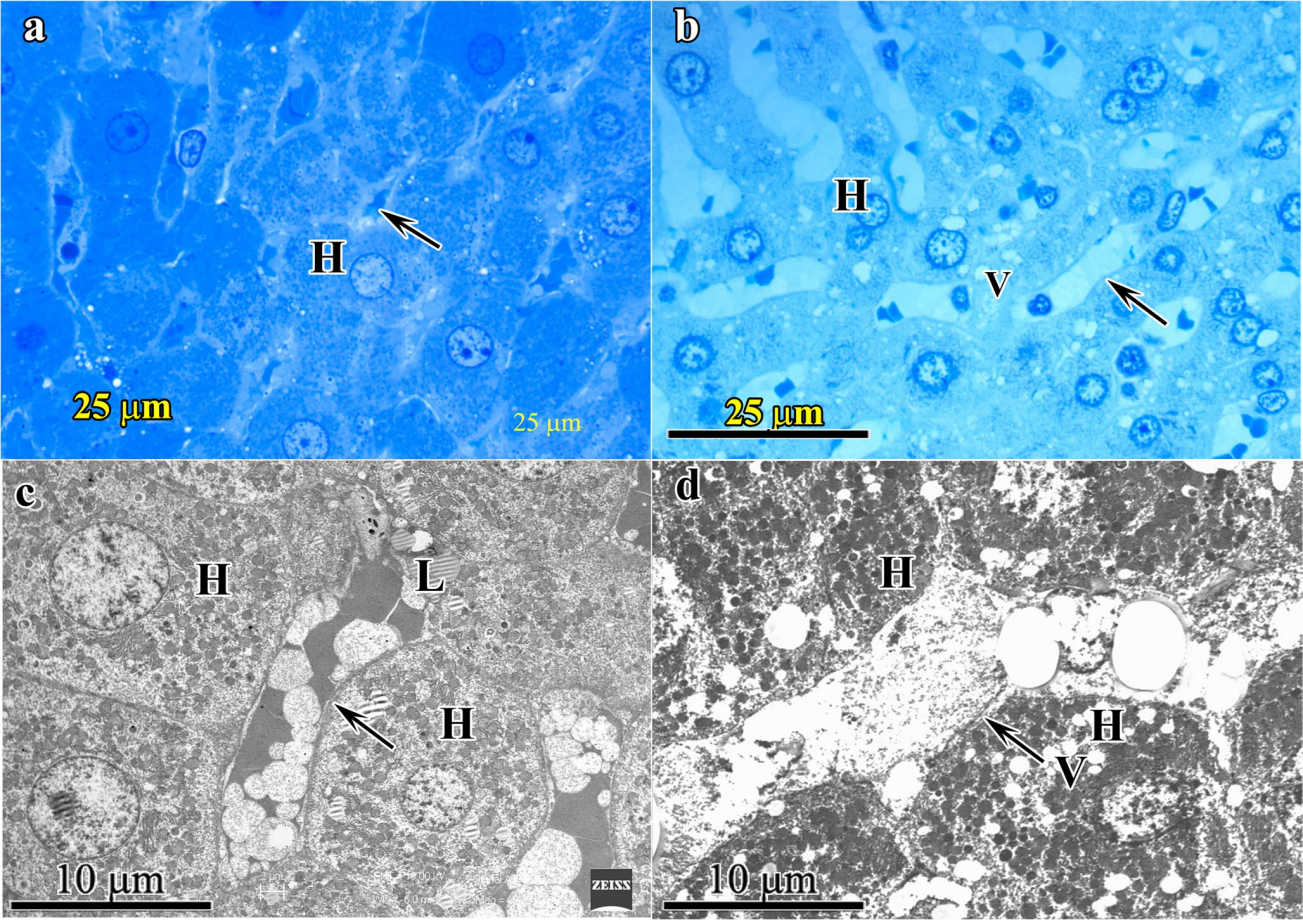
Liver-Elderly standard diet (Group-3) group and Elderly high protein diet (Group-4) Group Figures 2a and 2c, 2b-2d respectively. 2a: Group 3- In the semi- thin section hepatocytes (H) and sinusoids (→) are seen. 2b: Group 4- In semi- thin section hepatocytes (H), vacuoles (V) and dilated sinusoids (→) are seen. 2c: Group 3- In electron microscope lipid droplets (L) in hepatocytes (H), enlargement and stasis in the lumina of liver sinusoids (→) are seen. 2d: Group-4 Thin section of an enlarged sinusoid (→) and multiple vacuoles (V) of different sizes are present in hepatocytes reflecting steatosis (H). (2a and 2b: semi-thin section stained with toluidine blue; original magnification X100; 2c and 2d: thin sections stained with uranyl acetate-lead citrate; original magnification 1c and 1d: X5090)

Liver- Elderly groups fed with standard (Elderly SD (Group-3)) and high protein (Elderly HPD (Group-4)) diets: In Group-3 tissue samples, mild degenerative changes possibly related to aging were present when compared to the younger group. These include moderate swelling of hepatocytes with increased number of lipid droplets in their cytoplasm, enlargement and stasis in the lumina of liver sinusoids (Fig. 2a and c). In Group-4 degenerative changes were more pronounced. At light microscopic level hepatocytes having vacuoles of varying sizes cytoplasm and dilation of sinusoids was detected (Fig. 2b). Under electron microscope cytoplasmic swelling in hepatocytes and endothelial cells were observed (Fig. 2d).

**Fig. 3:**
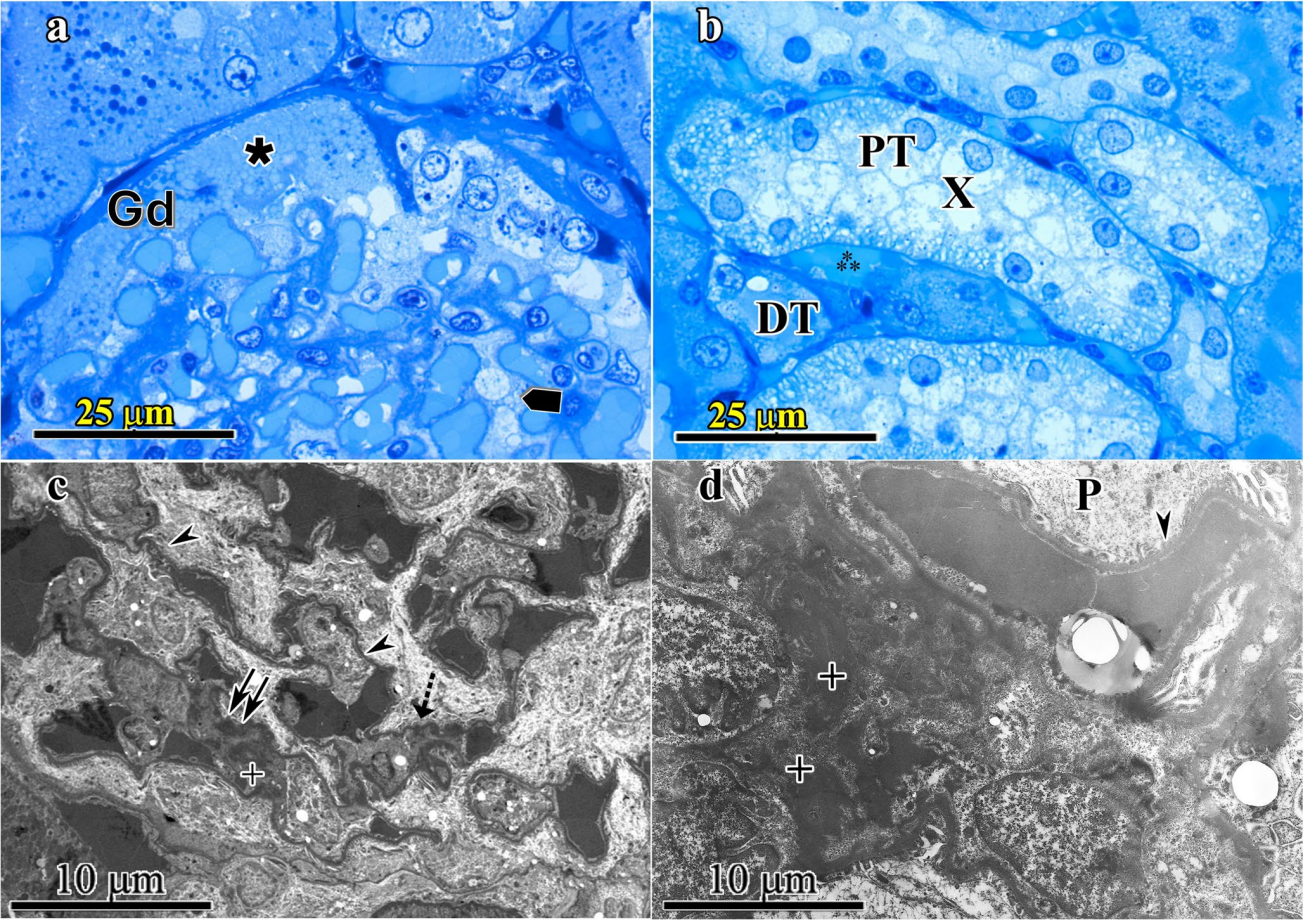
Kidney-Adult high protein diet group (Group-2) at light microscope (Figures- 3a and 3b) and electron microscope (Fig. 3c and d) were seen. 3a: In semi-thin section, glomeruli with degenerative changes (Gd) with enlarged glomerular capillaries, decreased Bowman’s space (✱) and hypertrophic mesangium including podocytes () are observed. 3b; In light microscopic level proximal tubule (PT) and distal tubules (DT) are distinguished. Tubular epithelial degeneration especially in proximal tubules (PT) and occlusion of tubular lumina with cellular debris (X) are present. Peritubular capillaries (⁂) were also dilated. 3c-3d; At electron microscopic level ondulation (⤏) and thickening (⇉)of glomerular basal lamina, fused pedicels (➤) of podocytes (P) and mesangial deposits (+) are seen. (3a and 3b: semi-thin section stained with toluidine blue; original magnification X100; 3c and 3d: thin sections stained with uranyl acetate-lead citrate; original magnification 3c: X3630 and 3d: X3597)

Kidney- Adult SD (Group-1) and Adult HPD (Group-2): Glomerular components, all types of kidney tubules and the interstitium were examined in detail and all appeared normal in Group-1 samples. Under electron microscope glomerular filtration barrier components (pedicels of podocytes, filtration slits and diaphragm, glomerular basal lamina and mesangium) were all normal. In Group-2 samples distinctive changes were determined especially under electron microscope. At light microsocope glomerular capillaries were dilated, Bowman’s space is attenuated and mesangium appeared hypertrophied with swellings in the cells including podocytes as well (Fig. 3a). Tubular epithelial degeneration especially in proximal tubules and occlusion of tubular lumina with cellular debris were present (Fig. 3b). Under electron microscope glomerular pathologies including thickening of glomerular basal lamina, subendothelial deposition of granular material, swelling in parietal layer of Bowman’s capsule and enlargement of glomerular mass leading to occlusion of Bowman’s space could be counted. In some tubular epithelial cells darkening of the cytoplasm with condensation of their nuclei indicating pre-necrotic changes were also detected (Fig. 3c and d).

**Fig. 4:**
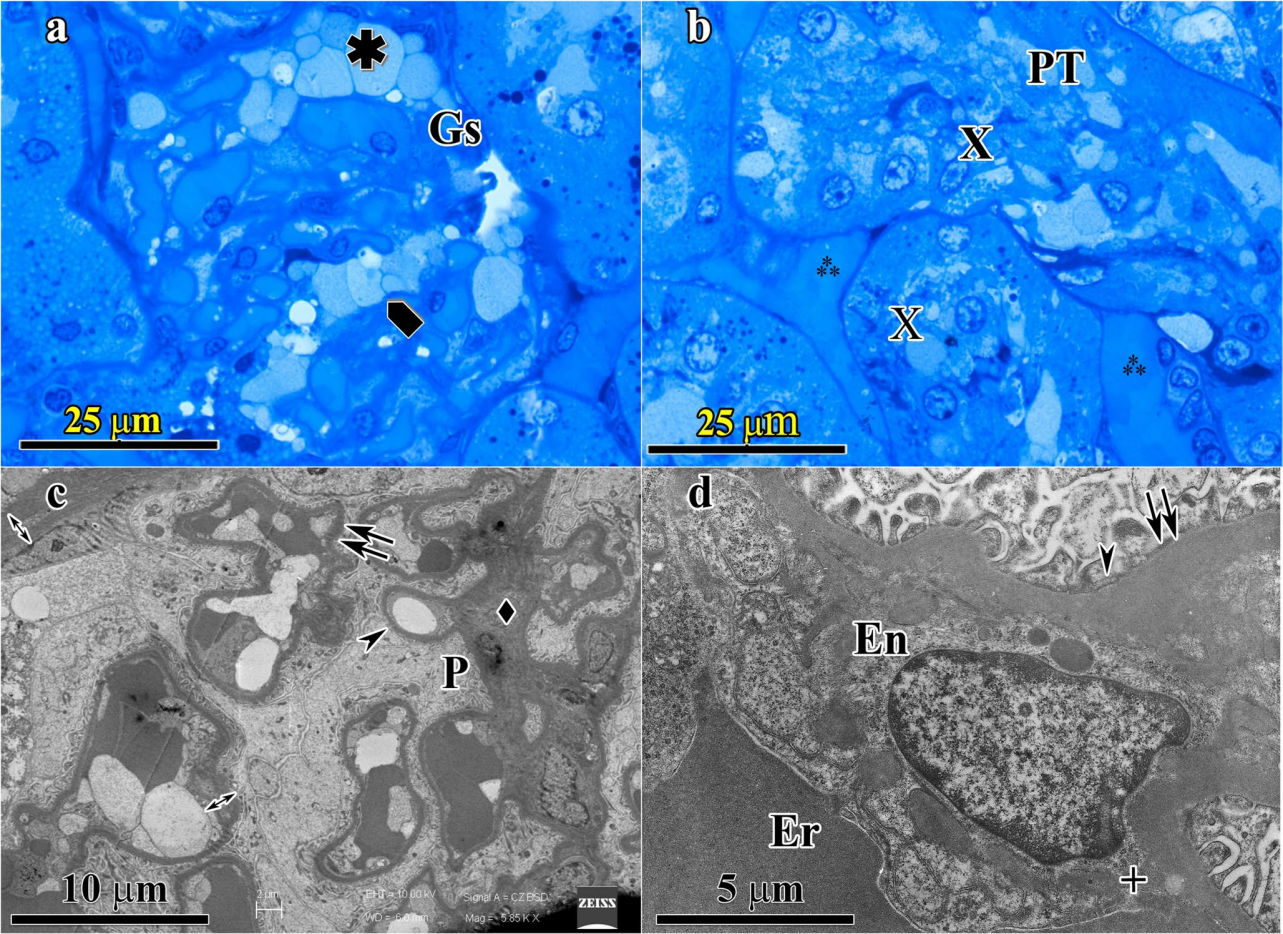
Kidney-Elderly high protein diet (Group-4) group. 4a: In semi-thin section, presence of dilated glomerular capillaries with diminished Bowman’s space (✱) suggesting changes towards glomerulosclerosis (Gs) is the most distinctive finding, with hypertrophic podocytes () also observed. 4b: Lumina of the proximal (PT) and distal tubules (DT) are usually crowded with pre-necrotic cellular debris material (**x**). Proximal tubular epithelial cells are also exhibiting degenerative structural changes. Dilated peritubular capillaries (⁂) are also observed. 4c-4d; At electron microscopic level, thickening (⇉) and lamination (separation) (↔) of glomerular basal lamina and mesangial deposits (+) are detected. Mesangial hypertrophy (⧫) and condensed glomerular mass are also distinguished. Hypertrophic podocytes (P) appear to occlude Bowman’s space. Pedicels also lost their normal structure some of which are fused (→). Er; Erythrocytes, En; Endothelial cell, Subendotelial deposites (+) are also seen. (4a and 4b: semi-thin section stained with toluidine blue; original magnification X100; 4c and 4d: thin sections stained with uranyl acetate-lead citrate; original magnification 4c: X5850 and 4d: X7750)

Kidney- Elderly SD (Group-3) and Elderly HPD (Group-4): In Group-3 samples only mild changes under light microscope like limited degenerative changes in some tubules were detected. The changes in Group-4 were much more significant when compared to the changes in liver. Presence of some sclerotic glomeruli was the most distinctive finding (Fig. 4a). Pre-necrotic changes in kidney tubular epithelial cells were also evident at light microscopic level. Lumina of the proximal and distal tubules were usually crowded with cellular debris material (Fig. 4b). Under electron microscope, thickening and lamination (separation) of glomerular basal lamina and mesangial deposits were seen. Mesangial hypertrophy, subendotelial deposits and sclerotic changes in glomerular mass were also detected. (Figure 4c and d).

### Biochemical results

Liver MDA levels, a marker of lipid peroxidation, differed significantly among groups. The Adult Standard Diet control group exhibited 14.4 ± 2.31 nmol/g, while the Adult High-Protein Diet showed a significant increase to 19.7 ± 2.43 nmol/g (*p* < 0.01 vs. Group 1). Similarly, the Elderly High Protein Diet group had elevated MDA levels (19.3 ± 2.14 nmol/g) compared to its control (Elderly Standart diets,: 13.4 ± 1.54 nmol/g, *p* < 0.01) (Table 1).

**Table 1:** Liver MDA and GSH concentrations. Values are mean ± SEM. The sample size of each group is 8

Groups	MDA (nmole/g)	GSH (nmole/g)
Adult SD- Group 1	14.4 ± 2.31	6.67 ± 0.93
Adult HPD- Group 2	19.7 ± 2.43*	6.45 ± 0.88
Elderly SD- Group 3	13.4 ± 1.54	5.53 ± 0.42 ^†^
Elderly HPD- Group 4	19.3 ± 2.14^*^	6.83 ± 0.78 ^‡^

^*^ Group 2 and Group 4 vs. their controls, ^†^ Group 3 vs. other groups, ^‡^Group 4 versus Group 1, *, †, ‡; *p* < 0.01

Liver GSH levels, indicating antioxidant capacity, were comparable between Adult Standart Diet (6.67 ± 0.93 µmol/g) and Adult high protein diet (6.45 ± 0.88 µmol/g). The Elderly standart diet group showed a significant decrease in GSH (5.53 ± 0.42 µmol/g, *p* < 0.01), whereas the elderly high protein diet group displayed higher GSH (6.83 ± 0.78 µmol/g, *p* < 0.01 vs. Adult standart diet). (Table 1).

Kidney MDA levels were significantly higher in Adult high protein diets (32.6 ± 4.8 nmol/g) compared to Adult starndart diet (25.6 ± 2.79 nmol/g, *p* < 0.01). Elderly high protein diets (28.6 ± 2.63 nmol/g) also showed elevated MDA relative to its control (Elderly standart diets, 26.02 ± 5.54 nmol/g, *p* < 0.01). (Table 2).

**Table 2:** Kidney MDA and GSH concentrations. Values are mean ± SEM. The sample size of each group is 8

Groups	MDA (nmole/g)	GSH (nmole/g)
Adult SD- Group 1	25.6 ± 2.79	6.03 ± 0.27
Adult HPD- Group 2	32.6 ± 4.8*	6.82 ± 0.84
Elderly SD- Group 3	26.02 ± 5.54	3.58 ± 0.28†
Elderly HPD- Group 4	28.6 ± 2.63*	5.75 ± 0.68‡

^*^ Group 2 and Group 4 vs. their controls, †Group 3 vs. other groups, ‡ Group 4 versus Group 3, *, †, ‡; *p* < 0.01

Kidney GSH levels followed a similar pattern. Adult standart and high protein diet groups were comparable (6.03 ± 0.27 µmol/g vs. 6.82 ± 0.84 µmol/g). The Elderly standart diet group exhibited a marked decrease (3.58 ± 0.28 µmol/g, *p* < 0.01), whereas the Elderly high protein diet group showed significantly higher GSH (5.75 ± 0.68 µmol/g, *p* < 0.01 vs. Elderly standart diet group). (Table 2).

### Statistical results

No significant differences were observed in the body weights among all groups. Liver and kidney weights also did not differ significantly between standard and high-protein diet groups in either adult or elderly rats. Although not statistically significant, a slight increase in body weight was noted in the elderly high-protein group.

Weekly measurements of food and water intake revealed a significant increase in food consumption in high-protein diet groups (both adult and elderly) compared to controls (*p* < 0.001). Similarly, water consumption increased over time in the high-protein groups (*p* < 0.001).

GDI scores differed significantly among the groups (Kruskal-Wallis H = 27.30, *p* < 0.001). Adult HPD and Elderly HPD rats exhibited significantly higher GDI scores compared to Adult SD and Elderly SD rats (*p* ≤ 0.004), whereas no significant differences were observed between Adult SD and Elderly SD (*p* = 1.0) or between Adult HPD and Elderly HPD (*p* = 0.171). These results indicate that a high-protein diet induces marked glomerular injury irrespective of age (Table 3).

**Table 3:** Glomerular damage index (GDI) and liver injury Scores. Values are mean ± SD. The sample size of each group is 8

Groups	GDI (0–4)	Liver Injury Score (0–4)
Adult SD- Group 1	0.00 ± 0.00	0.00 ± 0.00
Adult HPD- Group 2	2.50 ± 0.53*	1.13 ± 0.35*
Elderly SD- Group 3	0.25 ± 0.46†	1.63 ± 0.52†
Elderly HPD- Group 4	3.50 ± 0.53*‡	2.63 ± 0.52*‡

* Group 2 and 4 vs. their controls,† Group 3 vs. other groups, ‡ Group 4 vs. Group 1; *p* < 0.01

Liver injury scores also differed significantly among the groups (Kruskal-Wallis H = 26.84, *p* < 0.001). Adult HPD and Elderly SD rats showed higher scores than Adult SD rats (*p* ≤ 0.002), while Elderly HPD rats exhibited the highest injury scores, significantly exceeding those of Adult SD, Adult HPD, and Elderly SD rats (*p* ≤ 0.036). No significant difference was found between Adult HPD and Elderly SD (*p* = 0.316). These findings indicate that high-protein diet–induced liver injury is more pronounced in elderly rats (Table 3).

## Discussion

The rising prevalence of overweight and obesity, particularly in Western countries, has prompted increased attention to weight management. High-protein diets (HPDs), including low-calorie/high-protein and ketogenic approaches, as well as intermittent fasting, are commonly used for rapid weight loss [11, 12]. Although numerous studies have examined their benefits and potential adverse effects [5–7, 12–15], findings remain controversial, motivating the present investigation in rats to provide additional evidence. Such formulations are commonly employed in experimental models to simulate extreme high-protein conditions and to accelerate the manifestation of diet-induced effects. Therefore, while the model represents a supraphysiological condition, it provides valuable insight into the potential mechanisms and organ-specific responses to excessive protein intake. Liver and kidney tissues were assessed using light and electron microscopy, and tissue MDA and GSH levels were measured to evaluate oxidative stress and antioxidant capacity. Body weight, food intake, and water consumption were also monitored. The protein content used in this study (47.5%) exceeds typical human dietary intake; however, similar or even higher levels (up to 50%) have been applied in rodent studies for extended periods without overt tissue injury [3].

Although high-protein diets are widely associated with weight loss in both humans and experimental animals [3, 12, 13, 16, 17], no significant changes in body weight differences were observed in our model. This is likely attributable to the absence of caloric restriction, as protein-rich diets promote weight reduction primarily through reduced energy intake rather than protein content alone. Nevertheless, the lack of weight change does not diminish the relevance of our findings. Structural and biochemical alterations observed in the liver and kidneys suggest that even normocaloric HPD consumption may impose metabolic stress—particularly in aging organisms—independent of overt anthropometric changes. These observations can be contextualized within obesity and weight-loss frameworks, highlighting potential subclinical organ-level effects even in the absence of body weight changes. This finding parallels reports that high protein intake can affect renal hemodynamics and glomerular pressure independently of body weight [18, 19].

Histopathological analysis revealed that HPDs induced more pronounced degenerative changes in kidney tissues, including glomerular and tubular pathologies. Thickening and lamination of the glomerular basal lamina, subepithelial, subendothelial, and mesangial deposits, attenuated Bowman’s space, and tubular epithelial degeneration were observed. Liver tissue showed signs of steatosis in HPD-fed rats, particularly in the elderly group, whereas adult standard diet livers exhibited relatively normal features. These findings align with previous reports in laboratory animals [5, 20, 21], though some studies report minimal adverse effects of HPDs in animals with significant weight loss [3]. The semi-quantitative scoring results supported these observations, showing higher histological changes scores in HPD-fed rats—particularly in the elderly group—indicating that structural alterations were not only qualitatively evident but also quantitatively more pronounced with aging and high protein intake. These scores strengthen the interpretation that HPD-related injury progresses in an age-dependent manner rather than reflecting isolated or incidental microscopic findings.

Biochemical analyses demonstrated significantly elevated MDA levels in the liver and kidneys of HPD-fed adult and elderly rats, consistent with increased oxidative stress [15]. Hepatic and renal GSH levels were lower in elderly rats on a standard diet, reflecting age-related decline in antioxidant capacity [22]. Notably, elderly rats on HPD exhibited increased GSH levels, suggesting a compensatory metabolic response to oxidative stress, in line with prior observations [23].

Biochemically, increased MDA and variable GSH responses reflected differential oxidative stress handling across tissues and age groups. The notable rise in hepatic and renal GSH levels in elderly HPD rats may indicate an adaptive antioxidant response. One plausible mechanism involves the Nrf2 (nuclear factor-erityroid 2-related factor 2)/ARE (antioxidant response elements) pathway, a central regulator of redox homeostasis. Under oxidative challenge, Nrf2 dissociates from Keap1 (kelch like ECH associated protein 1), translocates into the nucleus, and activates transcription of detoxifying enzymes such as HO-1(heme oxygenase-1) and NQO1 (quinine oxidoreductase 1). In renal tissue, Nrf2 activation mitigates oxidative and inflammatory damage, while its deficiency exacerbates tubular injury. Although we did not directly assess Nrf2 or downstream targets, the GSH elevation observed here is consistent with an adaptive compensatory upregulation of the antioxidant defense system. Future studies quantifying Nrf2 expression or nuclear localization would clarify this mechanism [24–27].

Human studies generally report minimal changes in biochemical parameters following weight loss, although structural data are limited [17, 28–30]. HPDs should be used cautiously in individuals with pre-existing kidney disorders due to potential adverse effects on bone metabolism from high acid load [5, 6, 31]. Some evidence suggests that high-dairy HPDs may mitigate bone loss during weight reduction [14, 32]. Beyond weight loss, HPDs have been associated with delayed cancer initiation, improved glycemic control, and cardiovascular benefits, particularly with short-term use in overweight and obese individuals [13, 33, 34]. Conversely, some meta-analyses indicate potential cardiovascular risks [7], emphasizing the need for careful monitoring.

While human studies provide insights into clinical outcomes and potential risks, experimental data reveal underlying mechanisms and age-dependent effects that may not be fully captured in short-term human interventions. *E*merging research also implicates HPD-induced changes in gut microbiota composition in mediating systemic oxidative stress and metabolic outcomes. Altered microbial profiles and reduced short-chain fatty acid production under HPDs may indirectly affect hepatic lipid metabolism and renal oxidative load [35]. Additionally, in aging contexts, high protein intake can influence muscle proteostasis and sarcopenia risk—either beneficially via amino acid availability or detrimentally via oxidative imbalance [36, 37]. These multidimensional effects underscore that HPD outcomes depend strongly on both age and physiological context.

These findings collectively indicate that aging may modulate tissue responses to high-protein feeding through multiple interacting mechanisms. Age-related decline in mitochondrial efficiency and antioxidant capacity can exacerbate oxidative stress and impair cellular repair processes, leading to the more pronounced structural alterations observed in elderly rats. Reduced glutathione turnover and increased lipid peroxidation, as reflected by elevated MDA levels, may further contribute to hepatocellular and glomerular vulnerability. Moreover, previous studies have shown that aging alters amino acid metabolism and protein handling in both liver and kidney tissues, which could amplify the metabolic burden imposed by a high-protein diet. Taken together, these mechanisms may explain the greater susceptibility of aged organisms to diet-induced hepatic and renal injury observed in our study.

## Conclusion

High-protein diets can support weight loss and metabolic health, but caution is warranted, particularly in elderly individuals and those with pre-existing kidney conditions. Our findings indicate that aging significantly influences tissue susceptibility to dietary stress, emphasizing the need to consider age-dependent effects when designing protein-based nutritional strategies. Further studies are required to clarify the long-term consequences and safety of high-protein diets across the lifespan.

## Limitations

This study has some limitations. Only malondialdehyde (MDA) and glutathione (GSH) levels were evaluated to assess oxidative status. Although additional parameters such as catalase, superoxide dismutase, protein carbonyls, serum creatinine, and urea could provide a more comprehensive understanding of oxidative stress and organ function, these analyses were beyond the scope of the present study. Furthermore, the potential molecular mechanisms underlying the observed increase in GSH levels—such as Nrf2 pathway activation or other adaptive antioxidant responses—were not investigated. Future studies incorporating enzymatic and molecular markers will be valuable to clarify these mechanisms and confirm the compensatory responses suggested by the current findings.

## Supplementary Information

Below is the link to the electronic supplementary material.

Supplementary Material 1 (DOCX 34.5 KB)

## Data Availability

The datasets generated and/or analyzed during the current study are used in the paper.
